# Objective Cognitive Performance Modifies the Association Between Poor Sleep Health and Subjective Memory Decline

**DOI:** 10.1093/geroni/igaf122.1785

**Published:** 2025-12-31

**Authors:** Muhammad Thalil, Martin Sliwinski, Soomi Lee

**Affiliations:** The Pennsylvania State University, University Park, Pennsylvania, United States; The Pennsylvania State University, University Park, Pennsylvania, United States; The Pennsylvania State University, University Park, Pennsylvania, United States

## Abstract

Poor sleep health has been linked to subjective memory decline in older adults, yet the underlying mechanisms remain unclear. This study examined how objective cognitive performance may modify the relationship between sleep health and subjective memory decline in two cohorts of adults across adulthood. Two-wave longitudinal data were drawn from the National Health and Aging Trends Study (NHATS 2013 & 2014, n = 911; age 65+) and the Midlife in the United States Study (MIDUS 2004-2005 & 2013-2014, n = 2,421; age 33–84). A composite measure of sleep health, based on the self-reported Ru-SATED dimensions (regularity, satisfaction, alertness, timing, efficiency, duration), was used (higher score=poorer sleep health). Objective cognitive performance was assessed using episodic memory and executive function in MIDUS, with the addition of orientation in NHATS. After adjusting for baseline subjective memory and socio-demographic and health covariates, adults with poorer sleep health at baseline had higher odds of subjective memory decline in both the NHATS (OR: 1.21, 95% CI: 1.01-1.46) and MIDUS (OR: 1.24, 95% CI: 1.10-1.41) samples. In the NHATS only, the odds of subjective memory decline associated with poorer sleep health were more pronounced for older adults with worse cognitive performance at baseline (interaction p = 0.041). Middle-aged and older adults with poorer sleep health were at a higher risk of declining subjective memory. Those with both poorer sleep health and poorer objective cognitive performance were at particularly higher risk of subjective memory decline. These findings emphasize the importance of sleep-focused interventions for aging populations to help mitigate the risks of cognitive decline.

